# How do people with lived experience of Anorexia Nervosa and mental health professionals working with people with eating disorders conceptualise recovery?

**DOI:** 10.1186/s40337-025-01432-6

**Published:** 2025-10-31

**Authors:** Ana Julia Ferreira, Leda Blackwood, Manuela Martinez-Barona Soyer, Graeme Fairchild, Melissa Atkinson

**Affiliations:** Department of Psychology, University of Bath, Bath, UK

**Keywords:** Anorexia Nervosa, Eating disorders, Anorexia recovery, Qualitative, Lived experience perspectives

## Abstract

**Background:**

Understanding the reasons for recovery or relapse in individuals with Anorexia Nervosa (AN) is vital to improving post-treatment care. However, progress has been limited by a lack of consensus on defining recovery, with calls for including patient and clinician perspectives. This qualitative study explored how individuals with lived experience of AN and mental health professionals conceptualise recovery from AN, and the factors they perceived as contributing to or preventing recovery or relapse.

**Methods:**

We conducted three focus groups with people with lived experience of AN (n = 15 in total) and three focus groups and one individual interview with mental health professionals (n = 7). Data were analysed using reflexive thematic analysis.

**Results:**

We identified three themes. Theme 1 "Recovered, Yet Still Recovering" highlighted the ambiguity around what constitutes full recovery or whether it is possible and how people with lived experience and professionals’ views differed. Theme 2 “Disentangling Recovery and Weight Gain” highlighted different views between the groups on whether weight markers should be included in recovery definitions. Theme 3 “The Role of Others in Recovery: A Motivator or a Hinderance?” showed that people in one’s life can be the primary source of motivation to recover, but this is not sufficient for sustaining recovery and intrinsic motivators are needed.

**Discussion:**

Our study highlights that discrepancies between professional and lived experience definitions of recovery may be hindering therapeutic alliances, and that social support is crucial to promoting long term recovery. Additionally, we emphasise the importance of differentiating between cognitive, behavioural, and physical recovery in definitions.

**Supplementary Information:**

The online version contains supplementary material available at 10.1186/s40337-025-01432-6.

## Abstract (plain language summary)

Anorexia Nervosa is a serious eating disorder with high relapse rates after treatment. This study explored how people who have recovered from Anorexia and mental health professionals define recovery and what they believe makes it harder or easier. We held discussions with 15 individuals in recovery and 7 mental health professionals. We found that both groups agreed recovery is a long process, but they disagreed on whether full recovery is possible. Professionals saw recovery as managing symptoms, while people in recovery often felt that the presence of persistent disordered thoughts meant they were never fully recovered. Additionally, many people who have recovered from Anorexia felt that too much focus is placed on weight gain when defining recovery, whereas the professionals saw weight markers as very important. Additionally, social support was seen as important for motivation to recover, but not enough to sustain recovery alone. Participants suggested that finding personal goals and desires for recovery is important. Finally, participants highlighted the importance of having some support after leaving treatment, but they made clear that this should not take away from patients’ autonomy. Ultimately, this study sheds light on how best to define recovery which can help highlight important targets for aftercare following treatment.

**Supplementary Information:**

The online version contains supplementary material available at 10.1186/s40337-025-01432-6.

## Introduction

Anorexia Nervosa (AN) is a complex eating disorder (ED) characterised by restriction of energy intake and intense fear of weight gain [1]. A significant proportion of individuals relapse following treatment (37%; [8]), with the highest risk occurring in the first year [19]. More severe pathology, lower body mass index (BMI) at discharge from treatment, and younger age of ED onset have been associated with higher likelihood of relapse for those with AN [32]. Additionally, having comorbid diagnoses, especially depression and anxiety [8, 17], and lower motivation to recover [32, 37] also predict poorer outcomes. Social factors are also influential, with positive social support aiding recovery, whereas negative social experiences may hinder it [23]. However, differing definitions of ED recovery have led to difficulties comparing across projects and establishing the most important predictors of relapse [8, 32]. In order to establish a clearer understanding of factors that may lead to recovery or relapse, it is important to understand the definitions people are working with. Including lived experience and clinician perspectives is essential to shaping a more meaningful definition that is meaningful and shared by both groups.

Many studies have defined recovery using indicators such as a medically healthy weight (BMI > 18.5) and remission of disordered behaviours (e.g. [17]). While useful for standardisation, these criteria may overlook other equally important factors [38, 40]. Consensus is emerging to support Bardone-Cone et al.’s [3] definition of recovery that includes physical, behavioural *and* psychological recovery [38]. This definition differentiates full and partial recovery, enabling comprehensive investigations that account for the nuances of recovery [3]. Relatedly, people with lived experience of AN tend to focus more on subjective factors when defining recovery such as improved wellbeing and positive affect, decreased ED-related cognitions [9], and improved social connections [23, 30]. Importantly, this is not meant to supersede clinical measures of recovery but acknowledges a more holistic understanding [40]. Previous studies investigating lived experience views on recovery are becoming outdated, and new investigations may identify new insights in a post-covid world. Additionally, the perspectives of mental health professionals are not often accounted for when defining recovery. Doing so is crucial to acknowledge factors that may have previously been overlooked.

Building on this growing recognition of the value of lived experience perspectives, recent research has begun to explore the subjective experiences of recovery in greater depth. McCombie et al. [26] conducted a two-week qualitative daily diary study with ED-recovered patients, revealing the significant role of persistent ED-related thoughts despite outwardly non-disordered behaviour, which are often impacted by the social environment. However, the study did not specifically address how participants themselves defined recovery, and the daily diary method prevented deeper probing. Additionally, this study included individuals with diverse ED diagnoses. Focusing specifically on AN is important given its distinct features, and higher mortality [14] and relapse rates than other EDs [29]. Furthermore, incorporating both patients’ and clinicians’ perspectives on definition of recovery may highlight important points of contention. In an interview study, McDonald et al. [27] evaluated how service users and providers defined recovery, and their opinions on a proposed definition of recovery. They found that recovery is seen as an ongoing process by both groups and highlighted that definitions should be informed by lived experience perspectives. Additionally, they note hesitancy in including BMI markers in definitions of recovery, which calls for a deeper investigation of their usefulness. However, this paper did not investigate the factors these groups believe to affect recovery progressions, which we believe to be crucial in understanding how to prevent relapse. Making progress in supporting AN patients requires a clearer of understanding of how they and clinicians conceptualise recovery as well their conceptualisations of the factors affecting recovery. Hence, we conducted a qualitative focus group study with people with lived experience of AN, and mental health professionals who work with people with AN. We aimed to understand how these groups conceptualise recovery and what factors they believe may help or hinder it.

## Methods

### Reflexivity

The first author, a Latinx, cisgender female PhD student was responsible for recruitment, data collection, and led the data analysis. While she does not have a clinical background or personal history with AN, she has observed its impact on loved ones. She kept a reflexive journal and held weekly discussions with the research team, which included a female social psychologist, a male researcher specialising in youth mental health, a female clinical psychologist, and a female researcher specialising in eating disorders.

### Recruitment and participants

Eligibility criteria for lived experience (LE) participants included being 18 or older, residing in the United Kingdom, self-reported history of AN, and self-reported recovery. Interested participants completed the Eating Disorder Examination Questionnaire, a 28-item questionnaire providing a global score and four subscale scores (Restraint, Eating Concern, Shape Concern, and Weight Concern) based on responses rated on a 7-point scale, with higher scores indicating greater eating disorder psychopathology [12]. Those with global scores higher than 2.88 were excluded and signposted as this indicated that they had not reached full recovery according to Bardone-Cone et al. [3]. To be eligible as mental health professionals (MHP), participants had to be UK-qualified professionals who had worked with at least one individual with AN. Participants were recruited via social media, posters at university facilities, and emails to relevant groups.

Three focus groups were conducted with 15 individuals with LE, and three focus groups and one interview were held with seven MHP. Table 1 presents the participants’ characteristics. Names have been replaced with pseudonyms to protect participants’ identities. LE and MHP sessions were conducted separately and lasted 60–90 min. Participants were compensated with gift cards (£20; n = 21) or course credits (n = 1). Ethical approval was granted by the University’s Social Science Research Ethics Committee [reference: 3232–3746].

**Table 1:** Participant characteristics

Lived experience				
Pseudonym	Age range	Highest level of education completed	Gender	Race
Tina	18–22	A-level or equivalent	Cisgender female	Asian
Sam	18–22	A-level or equivalent	Cisgender female	White
Mandy	18–22	A-level or equivalent	Cisgender female	White
Mia	18–22	A-level or equivalent	Cisgender female	Asian
Sally	18–22	A-level or equivalent	Cisgender female	White
Rain	18–22	A-level or equivalent	Non-binary, assigned female at birth	Asian
Marie	18–22	Secondary school up to age 16	Cisgender female	White
Niamh	18–22	Undergraduate degree	Cisgender female	White
Laura	23–26	Undergraduate degree	Cisgender female	White
Ines	23–26	Undergraduate degree	Cisgender female	White
Aiofe	23–26	Post-graduate degree	Cisgender female	Mixed-race
Alisha	30–34	Post-graduate degree	Cisgender female	White
Stacey	30–34	Post-graduate degree	Cisgender female	White
Janet	46–50	Undergraduate degree	Cisgender female	White
Rose	50–55	Secondary school up to age 16	Cisgender female	White

Mental health professionals				
Pseudonym	Role	Years in current role	Gender	Race
Amanda	Clinical Psychologist	< 1 year	Cisgender female	White
Victoria	Clinical Psychologist	1–2 years	Cisgender female	Asian
Amber	Clinical Psychologist	3–6 years	Cisgender female	Black
Nicole	Occupational therapist	3–6 years	Cisgender female	Mixed-race
Elsa	Psychotherapist	1–2 years	Cisgender female	White
Darcy	Mental health support worker	< 1 year	Cisgender female	White
Devon	Assistant psychologist	< 1 year	Cisgender female	White

### Data collection

Focus groups were chosen to foster dynamic conversations and encourage more personal and sensitive disclosures [2, 18]. Due to issues with scheduling, one professional opted to be interviewed individually. Two LE focus groups were conducted in person, and all other sessions were conducted online on Microsoft Teams. This hybrid approach increased accessibility and allowed participation of a wider demographic. Online focus groups have been shown to facilitate engagement and produce findings comparable to face-to-face discussions [36].

The focus groups were guided by a semi-structured interview schedule asking about what recovery means to them, and what makes it easier or more difficult at different stages [see Additional File 1 for topic guide]. Participants were also asked to share their thoughts on mindfulness as a tool to aid recovery. This data was used to answer a subsequent research question on whether mindfulness practices can be used to aid recovery. It was therefore analysed and reported separately [13]. Participants were encouraged to express opinions freely and not be afraid to express disagreement. Poster boards and Padlet were available for participants to note down thoughts. These notes were included in the data for analysis. One participant subsequently emailed a list of thoughts she had during the discussion, which were also included in the data for analysis. All sessions were audio recorded and transcribed verbatim [31].

### Data analysis

Data were analysed using reflexive thematic analysis, using NVivo and following the six steps outlined by Braun and Clarke [4]. Codes were generated inductively, allowing findings to reflect participants’ views without being shaped by prior theories [4]. The researcher first familiarized herself with the data before beginning coding, identifying areas related to the research questions. The two participant groups transcripts were coded separately. Candidate themes were developed for each group, then compared to identify areas of agreement and disagreement. During the comparison stage, it became apparent that both groups had mostly highlighted similar topics but offered differing opinions on them. Overarching themes were identified and reviewed multiple times. The first author regularly met with the research team, who brought different knowledge and perspectives from clinical, neuro, and social psychology. We continued analysing the data while writing the manuscript to reflect the iterative process of thematic analysis.

The analyses were guided by a contextualist epistemological framework [25], and followed an experiential approach, meaning that it was concerned with capturing the reality of people’s lives [5]. A contextualist epistemology assumes that while participants’ experiences are meaningful and real, they are also shaped by broader social and cultural contexts, hence it acknowledges that knowledge is partially socially constructed [25]. Thus, while adopting an experiential approach that values participants’ lived experiences, we also recognise that these narratives are shaped by the context in which the study was conducted, prevailing social discourses, and the research team’s interpretations and backgrounds. During the analysis, we paid attention not only to what was said, but also to how ideas were framed.

## Results

**Fig. 1:**
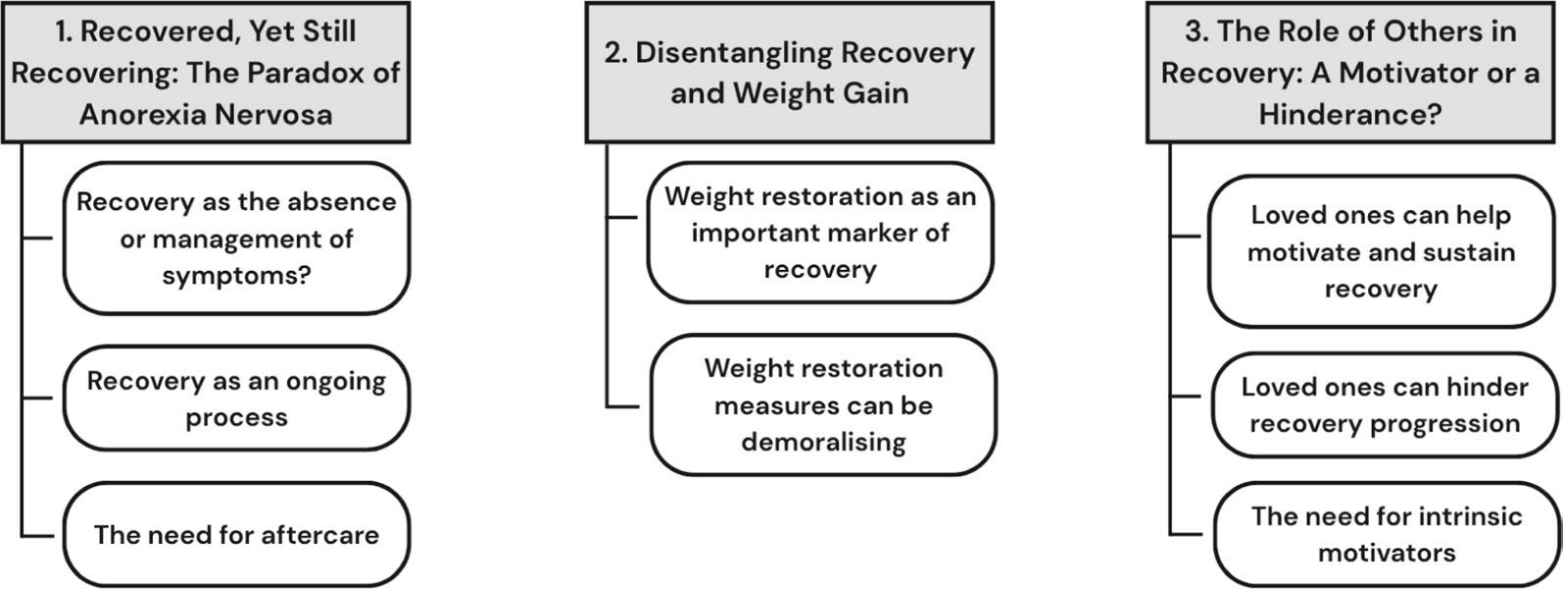
Thematic map

Three themes were identified regarding how people with lived experience (LE) and mental health professionals (MHP) conceptualise recovery from AN and the factors that contribute to recovery (see Fig. 1 for a thematic map). These were: (1) Recovered, Yet Still Recovering: The Paradox of Anorexia Nervosa; (2) Disentangling Recovery and Weight Gain; (3) The Role of Others in Recovery: A Motivator or a Hinderance?

### Theme 1—recovered, yet still recovering: the paradox of Anorexia Nervosa

This theme provides insight into how one’s belief regarding the possibility of full recovery depends on how one defines it. Participants in both groups distinguished cognitive and behavioural recovery, with “*the change in the behaviours definitely [coming] before trying to deal with your thoughts*” (Marie, LE participant). Cognitive recovery was considered crucial, with most participants reflecting that recovery would involve adaptive responses to disordered cognitions: *“you can recognise the thought but not engage in it*” (Amanda, clinical psychologist). Meanwhile, relapse was associated with “*listening to the thoughts and then acting on them*” (Mandy, LE Participant).

Participants were asked what recovery from AN means to them. Most MHP defined it not as the absence of thoughts or urges, but the ability to manage them in a way that allows for a fulfilling life.

It's not necessarily about […] completely getting rid of any thoughts or behaviours, but being able, kind of, to manage those thoughts in a healthy way, […] and enjoy their life without the Anorexia, like, stopping them in any way.—Darcy, mental health support worker

Darcy’s view underscores a functional definition of recovery—living well with residual symptoms. Victoria (clinical psychologist) summed it up saying “*it feels almost like a process like, for some people, [recovery] is like a verb rather than like a noun.*”

LE participants agreed that recovery was a process, with Laura (LE participant) likening it to a “*corkscrew*” where “*you have a rise, and you come back down, but when you come back down it allows you to go further up the next time.*” She thus acknowledges recovery as a non-linear process, which is in line with the MHP’s understanding. However, most LE participants promptly responded “*no*” when asked whether full recovery from AN is possible, citing the persistent cognitions as the reason. For them, ongoing effort meant they were always *in recovery* rather than being *recovered*.

LE participants reflected on different reasons for persistent ED-related thoughts, and why they consider themselves to be perpetually in recovery. Sally reflected on how her personality contributed to the development of her ED, and this is therefore a battle that she will constantly have to fight:

I think the fact that I, we, got into this situation in the first place-there’s gotta be some personality traits that facilitate that, and you can’t just change who you are as a person.—Sally, LE participant

Others cited constant exposure to harmful societal messages about food and weight. While most participants felt affected by these pressures, Janet described gaining distance from them:

I’m probably healthier than people, women particularly, in my life who’ve never had eating disorders. There’s so much societal stuff around - Oh, I don’t know, the clean eating thing nowadays, it’s frightening actually. But, […] I feel very trusting in my own relationship with food and eating because it’s come from experience and having worked out those issues and having to really do some questioning of the narrative around us—Janet, LE participant

Janet’s comments point to an important notion within AN recovery, which is the gendered societal standards in the West. In describing herself as having a healthier relationship with food than women “*who’ve never had an eating a disorder*”, she implicates gendered societal narratives around food and dieting and the need for women to question those narratives. From this perspective, the experience of recovering and the feeling that they will never recover is likely impacted by our participants’ (who were mostly cisgender women) gender identities. Stacey (LE participant) also mentioned that “*[her] relationship with [her] body post-anorexia and in recovery is probably better than it was before [she] actually got ill […] ‘cause you actually understand what your body does for you*”. Notably, these two participants were older than the others, suggesting this is something that may develop with age and time in recovery, although we did not collect data on this.

To summarise, LE participants conceptualised “full recovery” as a state of being not only free of symptoms but also free of any urges, triggers, or personality traits that may lead to a desire to reengage in unhelpful behaviours. They felt this standard was unattainable and consequently did not believe full recovery from AN exists. However, MHP believed that this process was part of, and did not preclude, full recovery.

The idea of ongoing recovery also raised questions regarding the role of aftercare. Participants felt that it would be helpful to access or offer some form of aftercare to help prevent relapse. However, MHP noted the difficulties of offering aftercare that involves healthcare professionals given the limited resources within the NHS:

I’m just thinking, like, how do you then manage, you know, keeping the caseload for a long time, and then the safeguarding and risk and all that?—Victoria, clinical psychologist

This view is notably tied to the UK context in which the study was conducted and may not be relevant elsewhere. More crucially, participants expressed concerns that some forms of aftercare could contribute further to the ambiguity around what constitutes recovery. Amanda (clinical psychologist) noted that it is not a good idea to keep people in care long-term “*because that kind of reinforces the idea that they can't cope on their own*”. This suggests that despite its appeal and potential benefits, continued regular support from ED treatment providers also has potential to inhibit one’s autonomy with their recovery. Notably, recovery here is being defined as the ability to “*cope on their own*” and thus providing extra care may be preventing recovery from being achieved until a later point when no such support is available. It is important to note that the idea of individualised coping is largely linked to Western female gender roles, whereby women are expected to be resilient and self-sufficient, and minimise any burden they may place on others [16]. This recommendation may therefore be a product of our participants’ primarily female gender identification. In accordance with Amanda’s point, LE participants spoke of self-help options such as “*a digital tool, maybe like an app that captures, or a way of being able to capture your thoughts*” (Stacey, LE participant) as viable for post-treatment support. These may be more feasible to deliver within current UK healthcare constraints and may enable greater autonomy. Yet, it is worth noting that these proposals assume that one has reached a threshold of recovery at the point of discharge and may not be applicable to those who leave treatment for other reasons (i.e. drop out).

### Theme 2—disentangling recovery and weight gain

LE and MHP tended to have different views on whether weight restoration should be a marker of recovery. MHP mentioned weight as being an important indicator of recovery:

We need a way to be able to quantify things. […] We need some sort of, you know, questionnaires, and weights […] on top of the qualitative thing, because of how important it is that they're discharged at the right time because of the really high risk in eating disorders.—Devon, assistant psychologist

Devon highlights the importance of weight restoration, particularly for safeguarding, acknowledging that more subjective measures of recovery are not enough. However, other MHP also acknowledged that this perspective can clash with service users’ opinions:

I think there's reluctance with the service users to admit that their weight is a kind of signal of how well they're doing. And this desire for like, you know, like, ‘my weight's not everything.’ Which, I agree with this completely, it’s not everything.—Darcy, mental health support worker

Darcy reflects a perspective that weight should not be the main component of recovery, as psychological and behavioural recovery may not be reflected in their weight. Other MHP pointed to systemic issues that lead to an overemphasis on weight:

Because of demand and stuff, it means that services will discharge when someone reaches a healthy weight […], and that can be then quite punitive for the patient. So, it's like weight is a way to get the care and stay in the service.—Amanda, clinical psychologist

Amanda highlights how this approach can disincentivize weight gain, as patients fear losing access to care. Darcy and Amanda highlight a dichotomy whereby weight can be overvalued in different ways: when a patient is psychologically recovered but remains underweight, or when a patient achieves weight restoration but is not yet psychologically recovered. LE participants agreed that there are issues with the current use of weight measures:

The only thing that was tracked was weight. In a way, it felt demoralizing because you would see it go up and you’d be like, ‘oh, I’ve done it! I managed to put on weight!’ And then you’d turn and like […] ‘Well, if you average it out, you haven’t actually put weight on.’ […] It kind of felt like they used it to beat you on the head a little bit.—Stacey, LE participant

The focus on weight, in Stacey’s view, overshadowed other meaningful progress. In fact, when asked to speak freely about recovery, LE participants did not bring up weight restoration. They only brought up weight when asked whether their healthcare team agreed with them on defining recovery, with a majority agreeing that professionals place too much emphasis on weight gain. There was agreement that the holistic picture of recovery is what matters to the LE participants, whether they were weight restored or not was not relevant:

I’ve always been quite underweight but what I class as being recovered wouldn't be me having a healthy BMI, it would be me being able to eat, like, comfortably, as opposed to [worrying] about like my weight or anything like that.—Rain, LE participant

As Rain illustrates, the desire to not think about their weight is so prominent that they would prefer not to worry about hitting any such markers. This may make sense when considering the amount of distress this measure can cause to someone suffering from AN, and a desire to distance themselves from it is perhaps unavoidable. It is notable that the way in which MHP and ED services frame weight measures may be unintentionally reinforcing a centrality of weight for their patients, even if this is not their goal. It has been shown that an overvaluation of weight is a key maintenance factor for AN [22], hence this recentering during treatment may be hindering recovery progressions. These different perceptions of the importance of weight restoration and its use as a marker of recovery highlight a key point of contention on how MHP and LE participants defined this process.

### Theme 3—the role of others in recovery: a motivator or a hinderance?

Participants were encouraged to discuss barriers and facilitators to recovery, and many noted their social environment as key. Some LE participants described the people in their lives as the key motivator for recovery.

I was eating because I thought, ‘oh my parents spend so much time, energy and money trying to get me better, so I have to eat.’ More than it was that I was recovered.—Mia, LE participant

For Mia, the desire to recover was not hers. Rather, she believed that it was the right thing to do for her parents. Aoife mentions that this type of motivation can also help sustain longer-term recovery:

I’m still with the same partner that I had when I was at my worst, and they used to have like physically carry me around the department to get to my lectures ‘cause I couldn’t do it. I can’t do that again. They’ve done that once and they deserve someone who’s actually able to look after themselves.—Aoife, LE participant

Additionally, Stacey mentioned wanting to recover so she did not negatively affect her daughter, and Janet noted that her patient-facing job within the ED field helps maintain motivation to stay in recovery. These reflections point to a lack of self-directed desire for recovery, leading to a reliance on external motivation. Rather than being concerned with their own health and wellbeing, participants seemed to find it easier to focus on how their ED affects others.

Whilst the social environment could provide motivation for recovery, some participants reflected on the potential for others to also be a hindrance.

I think other people can either be really good and really supportive or they can make things so much more difficult. And I don’t think people are really aware of the impact that they’re having necessarily.—Laura, LE participant

Laura highlights this influence, saying “*it can just be like all sorts of tiny things that can make [recovery] harder*” such as “*silly comments*” about food. Amber agreed, highlighting a hesitancy to discharge patients when they were aware of a lack of positive social support in their life:

It's also difficult to discharge people who are maybe going back to difficult environments where their eating disorder is triggered. […] I think we have to work a lot more on how to help people's wider networks to support and understand what's going on for the person now that they're being discharged.—Amber, clinical psychologist.

Amber’s view highlights the crucial nature of support systems, and the importance of psychoeducation and guidance on helpful ways to support loved ones through recovery. However, participants also made clear that it is important to find intrinsic motivators to sustain recovery.

You can want to recover for your family or for like external things, but if you don’t have that kind of strength inside of you and that desire inside of you to fully get better, well, then when it gets hard, you’re a lot more likely to just say like, ‘oh, screw it. I don’t care anymore. I don’t need to recover.’—Marie, LE participant

Marie acknowledges that social support may not always be there, and that to sustain motivation one must find it within themselves. MHP agreed, often stating that their patients who had wider life goals and an intrinsic desire to recover were less likely to relapse. This view may be linked to individualistic Western culture, whereby although social connections are valued, the primary focus is on the individual self. Hence, the perception that long-term recovery needs to be an individual process may be shaped by the Western context in which this study was done. Overall, one’s social environment is a key factor that affects recovery, and intrinsic motivators or broader life goals are crucial to helping sustain it.

## Discussion

We aimed to explore how people with lived experience (LE) of AN and mental health professionals (MHP) conceptualise recovery from AN, and what they consider helpful in promoting it. Our analysis identified the ambiguity surrounding what constitutes full recovery; contrasting views on the use of weight-related criteria in recovery definitions; and the powerful role of interpersonal relationships, while noting that they may not be sufficient. Participants’ accounts were shaped by broader discourses surrounding AN recovery. These include medicalised understandings of weight restoration as a key marker of recovery, individualistic ideals of personal responsibility, and gendered expectations about self-sufficiency.

Our findings highlight that emphasis should be placed on the distinctions between cognitive, behavioural, and physical recovery. Both LE participants and MHP believed that behavioural and cognitive recovery are distinct, with the latter taking longer to occur. This aligns with Bardone-Cone et al.’s [3] definition of recovery. MHP framed full recovery as managing residual symptoms adaptively, while LE participants often viewed full recovery as the complete absence of disordered thoughts, concluding that it does not exist. Bardone-Cone and colleagues’ [3] definition of full recovery, however, does not expect a complete absence of psychological symptoms but rather a similar level to community norms. Whilst our data cannot speak to what underlies LE participants’ perceptions, wider evidence points to perfectionism and cognitive rigidity, traits frequently associated with AN [24, 28], driving unrealistic expectations around recovery. Our participants acknowledged that those without a history of EDs also tend to have distorted ideals related to food and their bodies, which highlights that their expectations for themselves may be overly perfectionistic. Regarding weight criteria, LE participants noted that an overemphasis on weight gain as a marker of recovery was demoralizing and distressing. Our findings clearly demonstrate that definitions of recovery that are based purely on objective measures (i.e. [17]) are inappropriate, and holistic definitions of recovery that include subjective measures should always be adopted, which both of our participants group noted. This has also been highlighted in previous research [38, 40].

Ultimately, our findings highlight that different understandings between people with lived experience and medical professionals on what constitutes recovery may negatively affect recovery progressions. Darcy et al. [7] highlight that a discrepancy between professionals’ and patients’ beliefs about the importance of weight measures can disrupt the therapeutic alliance, which can affect patients’ recovery. It may therefore be crucial for practitioners to work with patients’ definitions. Recent investigations on weight-neutral care, which emphasizes eating for wellbeing and disentangling weight stigma, highlight positive outcomes in Bulimia Nervosa [10] and Binge Eating Disorder [11]. Though not yet established for AN, our findings suggest these approaches may improve therapeutic alliance and reduce distress. However, Waller and Mountford [39] note that regular weighing is a crucial component for the effectiveness of Cognitive Behavioural Therapy for EDs in helping challenge disordered beliefs and decrease anxiety around weighing via exposure. It is important to highlight that despite the strong feelings our participants seem to have against an overreliance on weight measures, they are seen as key markers of recovery in applied settings such as treatment centres due to treatment guidelines. Therefore, an epistemic injustice exists where MHPs and, perhaps more so, people with LE, are seen as less knowledgeable, and their perspectives are not sufficiently acknowledged when designing clinical guidance. Ultimately, more research is needed to determine how best to balance all perspectives, which could benefit from engaging with epistemic justice frameworks. Interestingly, our findings are in contrast with those of McDonald et al. [27], who found that service providers were also hesitant to include BMI markers in definitions of recovery. Their participants highlight the need to include flexible ways to measure weight restoration rather than focusing only on BMI, which should be explored in further research.

Furthermore, one’s social environment was also found to be a key influence on recovery. In accordance with previous research [23], social support was seen as crucial to helping or hindering recovery. Participants described being driven to get better by the impact of their illness on others, though they acknowledged that this alone might not sustain recovery and intrinsic motivators are needed. Previous research has shown that higher intrinsic motivation at the start of inpatient treatment predicts better treatment outcomes, which was not true for extrinsic motivation [34]. Future research should aim to explore the role of intrinsic and extrinsic motivators in post-treatment recovery. Additionally, as we note that these perspectives may be affected by individualistic Western biases, future research could benefit from a cultural analysis of this phenomenon. Additionally, as noted, participants highlighted being affected by harmful societal messages related to weight and diet. It is therefore crucial that these factors are addressed. Indeed, our findings highlighted that more aftercare support is needed following discharge from treatment, but it is important that this can help promote autonomy. Self-guided tools may be important focus of further exploration, as they can help maintain autonomy while providing support. Specifically, they should include social components where possible (i.e. psychoeducation for family and friends) and help patients disentangle from harmful societal narratives around food, weight, and shape. Media literacy interventions have shown preventative effects for EDs [20], and future work should examine whether these tools could support long-term recovery. Moreover, we stress a need for societal-level interventions to decrease the prevalence of such harmful messaging.

Finally, although previous works have highlighted the ongoing nature of recovery [27], our findings make clear that ED cognitions were seen to be a primary source of distress post-treatment. Cognitive Behavioural Therapy-based aftercare interventions have yielded promising results for relapse prevention in this population (i.e. [15]), and merit further study. Additionally, the use of mindfulness may be helpful in teaching patients to accept their thoughts nonjudgmentally and not act on them, improving emotional regulation [6]. It may also help them accept the continued presence of disordered cognitions and recognise that it does not take away from their recovery. There is some support for the use of mindfulness in EDs [33], but more work is needed to establish its effectiveness, and if it can be used as an aftercare tool.

## Limitations

Firstly, our sample consisted primarily of highly educated Caucasian females, which decreases the range of perspectives explored here. Our homogenous sample is largely due to the convenience sampling methods used, and the limited resources to recruit further for this study. This limited diversity may have constrained the emergence of alternative understandings of recovery that are shaped by intersections of race, age, gender identity, or socioeconomic context, and may inadvertently reinforce the marginalisation of experiences that fall outside the normative frame. Future studies would benefit from targeted recruitment of those from marginalised and underrepresented groups (e.g. ethical and racial minorities, men, those who did not have access to ED treatment, etc.) to investigate how structural oppression intersects with ED recovery. For instance, Lee et al. [21] examined how recovery from AN is experienced by a sample of Taiwanese women, highlighting how cultural conflicts between traditional values and Western influences play a role in the development and maintenance of their disorder, and how their integration is necessary for recovery. The emergence of more studies like this would allow for a more holistic view of recovery that is able to extend beyond the majority perspective and significantly improve our understanding of this phenomenon. We acknowledge that conceptualisations of recovery must be understood in relation to structural conditions that shape access to care, but that we were unable to do so in this project due to our homogenous sample.

Second, the participants worked or experienced treatment in the United Kingdom, meaning international variation in ED treatment is not captured. Comparative and international studies are needed to understand how definitions of recovery may differ between countries and cultures (e.g., would people living in more collectivist societies still emphasise the importance of intrinsic motivators in achieving sustained recovery?). Third, focus groups, while valuable for exploring shared experiences, can introduce groupthink, possibly suppressing dissenting views. Fourth, we did not gather information on when participants were diagnosed, how long they had been out of treatment, or what type of treatment they received, limiting our ability to explore how these variables influenced their perspectives. Fifth, our MHP sample skewed toward early-career professionals, and future work should include more experienced clinicians.

Finally, we acknowledge a need for co-produced research in this field which includes people with LE at all stages of the research, including setting of research priorities, study design, data interpretation, and dissemination. This can increase the impact and quality of the research, ensuring it is aligned with stakeholder perspectives. Most importantly, this can help decrease epistemic imbalances amongst those with different forms of expertise [35].

## Conclusion

This study provides a novel qualitative exploration of both patient and clinician perspectives on recovery from AN, identifying points of alignment and divergence. We find that discrepancies between professional and lived experience definitions of recovery, especially with regards to the focus on of weight restoration, may be hindering therapeutic alliances and ultimately recovery progressions. We urge clinical decision makers and researchers to show greater attempts at including LE perspectives in definitions of recovery. Furthermore, our findings highlight a crucial need for aftercare support following treatment, especially that which can be self-guided and is able to target remaining ED cognitions that may hinder recovery progressions. Ultimately, we hope these findings can be used to improve current clinical practice and future research to help promote recovery for those with AN.

## Supplementary Information

Additional file 1.

## Data Availability

All data created during this research is stored in the University of Bath Research Data Archive at 10.15125/BATH-01541. Due to nature of the consent obtained, access may only be granted to researchers from the original project team or those who have entered into a data sharing agreement with the project team.

## Abbreviations

AN: Anorexia Nervosa
ED: Eating disorder
BMI: Body Mass Index
LE: Lived experience
MHP: Mental health professional
