# Supplementary material for: How do people with lived experience of Anorexia Nervosa and mental health professionals working with people with eating disorders conceptualise recovery?
Source: J Eat Disord. 2025 Oct 31;13:247. doi: 10.1186/s40337-025-01432-6 (PMC12577377; doi:10.1186/s40337-025-01432-6)
Supplement: Supplementary file 1 — Additional file 1. [file 40337_2025_1432_MOESM1_ESM.docx]

**Supplementary materials**

**Supplementary Table 1**

*Focus group topic guides and suggested prompts for lived experience*

| **Guiding question** | **Suggested follow up prompts** |
| --- | --- |
| What does the concept of recovery from Anorexia mean to you? | When did you feel like you were recovered?  Do you think there is such a thing as full recovery? |
| What does the concept of relapse mean to you? | When would you feel like you had relapsed? |
| Do you think there is a general consensus on these definitions amongst people who have had anorexia? | Would doctors agree with these definitions? |
| What are some things that made recovery easier/harder when you first left treatment? | Any people/social environments that made a difference? Any specific thoughts or ways of thinking?  What were the major challenges? |
| What are some things that made recovery easier/harder a few months after leaving treatment? |  |
| What kinds of things, if any, do you think still impact you now? |  |
| What are some things you notice make relapse more likely? |  |
| What are some things you notice have made you resilient to relapse? | For instance, coping strategies, things learned in therapy, specific people |
| When do you think relapse is most likely to happen? |  |
| Does anyone have any experience they would like to share of going back to treatment and how they felt? |  |

**Supplementary Table 2**

*Focus group topic guides and suggested prompts for mental health professionals*

| **Guiding question** | **Suggested follow up prompts** |
| --- | --- |
| What does the concept of recovery from Anorexia mean to you? | When do you consider someone to be recovered?  Do you think there is such a full recovery? |
| What does the concept of relapse mean to you? | When would you feel like a client had relapsed? |
| Do you think there is a general consensus on these definitions amongst mental health professionals? | Would your clients agree with these definitions? |
| What is important to look at when considering whether to discharge a patient from treatment? | Are they recovered at this point? |
| What kinds of things seem to be important for recovery when clients are first discharged from treatment? | Any people/social environments that make a difference? Any specific thoughts or ways of thinking?  What are some typical major challenges? |
| What kinds of things, if any, do you think still impact them once they are considered recovered? |  |
| What support does your service provide, if any, following discharge? Does this strategy change as clients progress in their recovery? | Do you think you could do more? If so, what? |
| What are some things you notice about clients who return to treatment after relapse? | What are the key differences between those who relapse and those who don’t?  How does treatment change for a returning patient? |
| What are things that you have noticed make people more or less likely to relapse? |  |
| When do you think relapse is most likely to happen? |  |
